# The effectiveness and safety of Tanreqing Injection combined with ganciclovir on the treatment of respiratory syncytial virus pneumonia in children: A protocol for systematic review and meta-analysis of randomized controlled trials

**DOI:** 10.1097/MD.0000000000022356

**Published:** 2020-09-18

**Authors:** Wei-Jun Zhu, Xuan Zhou, Juan Cao, Yu Shi

**Affiliations:** a Department of Pediatric, Haikou Hospital of the Maternal and Child Health, Guoxing Avenue; b Department of Pediatric, Hainan Modern Women and Children's Hospital, Qiongzhou Avenue, Qiongshan District, Haikou, Hainan, China.

**Keywords:** cochrane handbook for systematic reviews of interventions, effectiveness and safety, ganciclovir, respiratory syncytial virus pneumonia in children, systematic review and meta-analysis, tanreqing injection, traditional Chinese medicine

## Abstract

**Background:**

Based on the Theory of Traditional Chinese Medicine, this study systematically evaluated the effectiveness and safety of Chinese medicine preparation Tanreqing injection combined with ganciclovir on the treatment of respiratory syncytial virus pneumonia in children, and provided new ideas and methods for the treatment of respiratory syncytial virus pneumonia (RSVP) in children. At the same time, it also studies the effectiveness and safety of the combination of Chinese and Western medicine on the treatment of related diseases from the direction of evidence-based medicine.

**Methods:**

The relevant literature was searched by the computer in the electronic network databases, the retrieved databases include Chinese database and English database, English database includes PubMed, Cochrane Library, Embase and Web of Science. Chinese database includes: CNKI, SinoMed, WangFang Date, VIP and other networks electronic full-text database, conducting a randomized controlled trial of Tanreqing Injection combined with ganciclovir (study group) and ganciclovir alone (control group) on the treatment of RSVP in children and the retrieval time limit is set from the establishment of each database to July 1, 2020. According to the inclusion and exclusion criteria, the literature is independently searched and screened by 2 researchers, and conducting the full-text retrieval and evaluation of the research to be included, and extracting the information and checking it after reading the full-text; In case of disagreement, a third researcher will be invited to participate, and the decision is made after discussion by the 3 researchers. They were using the bias risk assessment tool provided by the Cochrane Handbook for Systematic Reviews of Interventions 3.0.2 to evaluate the selected literature. They were using RevMan 5.3 statistical software to conduct statistical analysis.

**Results:**

This study will be carried out in full accordance with the steps of systematic review as required in the Cochrane Handbook for Systematic Reviews of Interventions. All research results will be published publicly in international academic journals with peer review.

**Conclusion:**

After the meta-analysis of Tanreqing injection combined with ganciclovir on the treatment of RSVP in children, this paper will give a scientific and objective judgment on the effectiveness and safety of the combined use of Chinese and Western medicine on the treatment of RSVP in children, to provide evidence-based medical evidence for the clinical application, effectiveness and safety of Chinese and Western medicine combined on the treatment of RSVP in children.

**PROSPERO registration number:**

OSF platform, registration number: j2bz5.

## 1 Introduction

Respiratory syncytial virus (RSV) is 1 of the most common pathogens of respiratory tract infection in infants and children. Clinically, the virus often causes respiratory syncytial virus pneumonia (RSVP) or bronchiolitis. RSV is a single-stranded RNA virus belonging to the genus pneumonia virus of Paramyxoviridae, and its genome encodes 11 proteins, which are mainly caused by perforin G and F.^[1–4]^ RSV is mainly transmitted by droplets, and it can also be transmitted by indirect contact with the substances contaminated by children's respiratory secretions. The main pathological changes are as follows: congestion and oedema of nasal and pharyngeal mucosa, necrosis and denudation of bronchial mucosa, degeneration of alveolar epithelial cells and substantial necrosis and collapse of alveolar.^[5,6]^

Pneumonia is a common and frequent disease in paediatrics, which caused by RSV infection, and it is the primary cause of hospitalized infants and children. Because of its acute onset, rapid progress and severe illness, the Ministry of Health of our country divides it into 1 of the 4 major pediatric diseases, and it is also listed as 1 of the 3 important global childhood diseases by World Health Organization.^[7]^ The studies have shown that most of the pneumonia in infants and children, especially those within 6 months, is caused by a virus infection, and the most common pathogens are RSV, influenza virus, adenovirus, and so on. The infection caused by RSV is the first. RSV has 2 subtypes, A and B, and China is dominated by A subtypes. Meanwhile, the infection rate of RSV also increased year by year, which has become 1 of the diseases that can seriously threaten the health of the infants and children.^[8,9]^ Because the development of infant immune system is not perfect and the secretion of immunoglobulin A secretion in respiratory tract mucosa is insufficient, so it is likely to be infected with pneumonia caused by RSV. The main clinical manifestations are fever, cough, shortness of breath, nasal oedema, phlegm between the throat. RSV infection can also induce allergies and asthma, aggravate asthma, and even induce heart failure and other critical conditions. Therefore, it is suddenly important to choose a suitable and effective treatment.^[10]^

At present, there is no specific treatment for viral infection, and symptomatic and supportive treatment is still the main treatment for neonatal RSV pneumonia. For example, oxygen inhalation, salbutamol or glucocorticoid atomization therapy can be selected for childhood asthma, shortness of breath. Antiviral drugs (ribavirin, ganciclovir, etc.) can be used for RSV itself. However, due to the lack of specific drugs, the current treatment measures have not achieved excellent clinical effect.^[11]^ Therefore, the prevention and treatment of RSV infection have gradually attracted more attention from medical workers. In the treatment of viral infectious pneumonia, Chinese medicine has a long history, traditional Chinese medicine treatment of viral infectious pneumonia can significantly improve the clinical manifestations, and shorten the course of the disease and can also reduce the side effects of antiviral drugs and other advantages. Chinese medicine mainly has the effect of inhibiting virus replication and proliferation in vivo, regulating the immune function of the body, and it has the obvious advantages and potential for the development of respiratory diseases caused by RSV infection, which is worthy of clinical promotion and in-depth study.^[12]^

As a traditional Chinese medicine preparation, Tanreqing injection is composed of Huangqin (*Scutellariae Radix*), Xiongdanfen (*Bear gall powder*), Shanyangjiao (*Caprae hircus cornu*), Jinyinghua (*Flos lonicerae*) and Lianqiao (*Fructus forsythiae*). Among them, principle drug is *Scutellariae Radix*, which has the effects of purging fire and detoxification, clearing heat and dampness. *Bear gall powder* has expectorant, antitussive, bacteriostasis, detoxification, antispasmodic and other effects, and the effects of *Caprae hircus cornu* and *Cornu saigae tataricae* have a certain similarity, which can clear heat and detoxify. *Flos lonicerae* and *Fructus forsythiae* have antiviral effects. The combination of various drugs has the effects of detoxifying, resolving phlegm, and it is often used in the treatment of an acute bronchitis, acute attack of chronic bronchitis and upper respiratory tract infection.^[13]^ In recent years, it has been reported that Tanreqing injection can effectively protect cells from the damage of RSV, and it can directly inactivate or inhibit of RSV virus by changing the structure of the virus and the number and structure of viral receptors on the cell membrane.^[14]^ Ganciclovir is a commonly used antiviral drug in the clinic. When ganciclovir enters the host cell, it can be phosphorylated by the sensitive virus to guide 1 or more cell kinases, and the concentration of ganciclovir is high in the cell, so it can play a competitive role in inhibiting viral DNA polymerase. Ganciclovir is a guanine homologue, which is commonly used in the prevention and treatment of cytomegalovirus infection in patients with immune deficiency. Ganciclovir can inhibit the synthesis of viral DNA, to cure the diseases thoroughly. Ganciclovir has fewer adverse reactions, which is mainly through glomerular filtration to prototype excretion. After injection, its drug plasma clearance rate and half-life are short, and it will not cause excessive residue and other conditions.^[15]^ Chinese clinicians have combined Tanreqing injection and ganciclovir, to treat children with RSV pneumonia, but there are few related research reports. At present, there are disputes about the safety and effectiveness of the combination of Chinese and Western medicine in the treatment of children with RSV pneumonia.

Based on the Theory of Traditional Chinese Medicine, this study systematically evaluated the effectiveness and safety of Chinese medicine preparation Tanreqing injection combined with ganciclovir on the treatment of RSVP in children, and used evidence-based medicine to provide new ideas and methods for the treatment of RSVP pneumonia in children.

## 2 Objectives

The purpose of this study is based on the theory of traditional Chinese medicine, and systematically evaluates the effectiveness and safety of Chinese medicine preparation Tanreqing injection combined with ganciclovir on the treatment of RSVP in children.

## 3 Methods

### 3.1 Study registering and reporting

The protocol of our meta-analysis has been registered on the OSF platform (OSF, https://osf.io). The registration number was j2bz5.

### 3.2 Eligibility criteria

#### 3.2.1 Type of study

This study selected a randomized controlled trial of the Chinese medicine preparation Tanreqing injection combined with ganciclovir on the treatment of RSVP in children. The selected literature must follow the 3 basic principles of the randomized controlled trial, namely, control group, randomization group and blind trial, and the language limit of the literature is Chinese or English.^[16]^

#### 3.2.2 Types of participants

The participants must meet pre-established inclusion and exclusion criteria.

##### 3.2.2.1 Inclusion criteria

(1) The diagnostic criteria of RSV pneumonia in neonates refer to Practical Neonatology (4th Edition);^[17]^
(2) Clinical manifestations: cough, snivel, sneezing, phlegm, wheezing, wheezing or moist rales in auscultation of lung;
(3) Chest X-ray manifestations: double lung scattered in small patch shadow, the texture of double lung thickening or excessive expansion and stripe shadow, emphysema;
(4) Blood routine examination: the count of peripheral blood white blood cell may be normal or slightly reduced, the proportion of lymphocyte is relatively increased;
(5) A disposable sterile sputum suction tube was used after tracheal intubation on the day of admission to the hospital, to absorb some of the secretions of the larynx under pressure. Direct immunofluorescence detection is used, which indicates that RSV-Ag is positive, and there are no extrapulmonary complications. No serious bacterial infections;
(6) The medical history of sick children is complete.

##### 3.2.2.2 Exclusion criteria

(1) Children with severe extrapulmonary complications of RSV infection complicated with respiratory failure;
(2) Children with other severe primary diseases and children with mental illness
(3) Children who allergy to the drugs used in this study;
(4) Children with poor compliance and unable to complete the treatment according to the regulations;
(5) Children whose curative effect cannot be determined, or the data is incomplete, which affects the efficacy or safety evaluation;
(6) Animal experiment or clinical pharmacokinetic studies or reviews;
(7) Repetitive literature;
(8) Unable to get valid data.

#### 3.2.3 Types of interventions

##### 3.2.3.1 Interventions

Tanreqing injection + ganciclovir (dose, course of treatment, the dosage form of drugs, there is no limit to the manufacturers of drugs).

##### 3.2.3.2 Comparison

Ganciclovir (dose, course of treatment, the dosage form of drugs, there is no limit to the manufacturers of drugs).

### 3.3 Types of outcomes

#### 3.3.1 Main outcome indicators

(1) The total effective rate (cure rate + significant rate + effective rate);
(2) Hospitalization time;
(3) Antipyretic time;
(4) Cough disappearance time;
(5) Pulmonary moist rales disappearance time;
(6) Chest X-ray inflammatory absorption time;
(7) The occurrence of adverse reactions.

#### 3.3.2 Secondary outcomes

##### 3.3.2.1 Tidal breathing pulmonary function

(1) Forced vital capacity;
(2) Vital capacity;
(3) Peak expiratory flow;
(4) Forced expiratory volume in 1 second;
(5) Maximal voluntary ventilation

##### 3.3.2.2 The changes of T cell subsets

(1) CD_3_^+^;
(2) CD_4_^+^;
(3) CD_8_^+^.

##### 3.3.2.3 Serum inflammatory factors

(1) Interleukin (IL)-6;
(2) IL- 8;
(3) IL-18.

### 3.4 Searching strategy

The literature acquisition of this study adopts the method of computer retrieval network database and manual retrieval of relevant historical materials, computer retrieval of the foreign database are PubMed The Cochrane Library Embase and Web of Science; the retrieval of Chinese database are CNKI, WangFang Date, SinoMed and VIP, and so on. The retrieval plan mainly adopts the combination of subject words and free words, among them, the Chinese terms are “Tan-re-qing-zhu-she-ye,” “Geng-xi-luo-wei,” “Hu-xi-dao-he-bao-bing-du-fei-yan,” “Fei-yan,” “Er-tong,” “Zhong-yi-yao,” “Kang-bing-du-yao,” and so on; The English terms are “Tanreqing Injection,” “Medicine, Chinese Traditional,” “Ganciclovir,” “Respiratory Syncytial Viruses,” “Respiratory Syncytial Virus Infections,” “Respiratory Syncytial Virus, Human,” “Pneumonia,” “Children,” “Antiviral Agents,” “Randomized controlled trial,” and so on. The retrieval language is set to Chinese and English, and the retrieval scope is set from the establishment of each database to July 1, 2020, while supporting the manual retrieval of relevant journals issued only in the paper version. The study selection flow diagram is shown in Table 1.

**Table 1:**
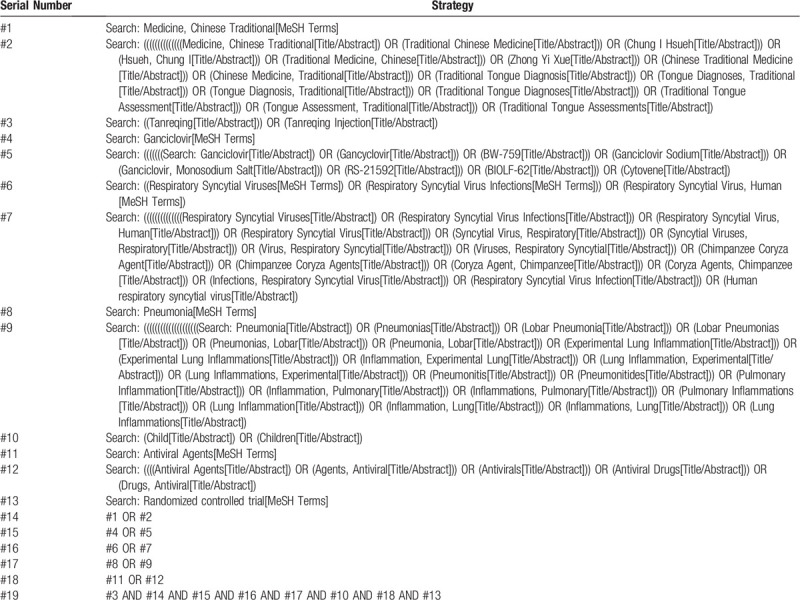
Search strategy for the PubMed database.

Table 1. Study selection flow diagram used in the PubMed database

### 3.5 Selection process and data extraction and management

**Figure 1:**
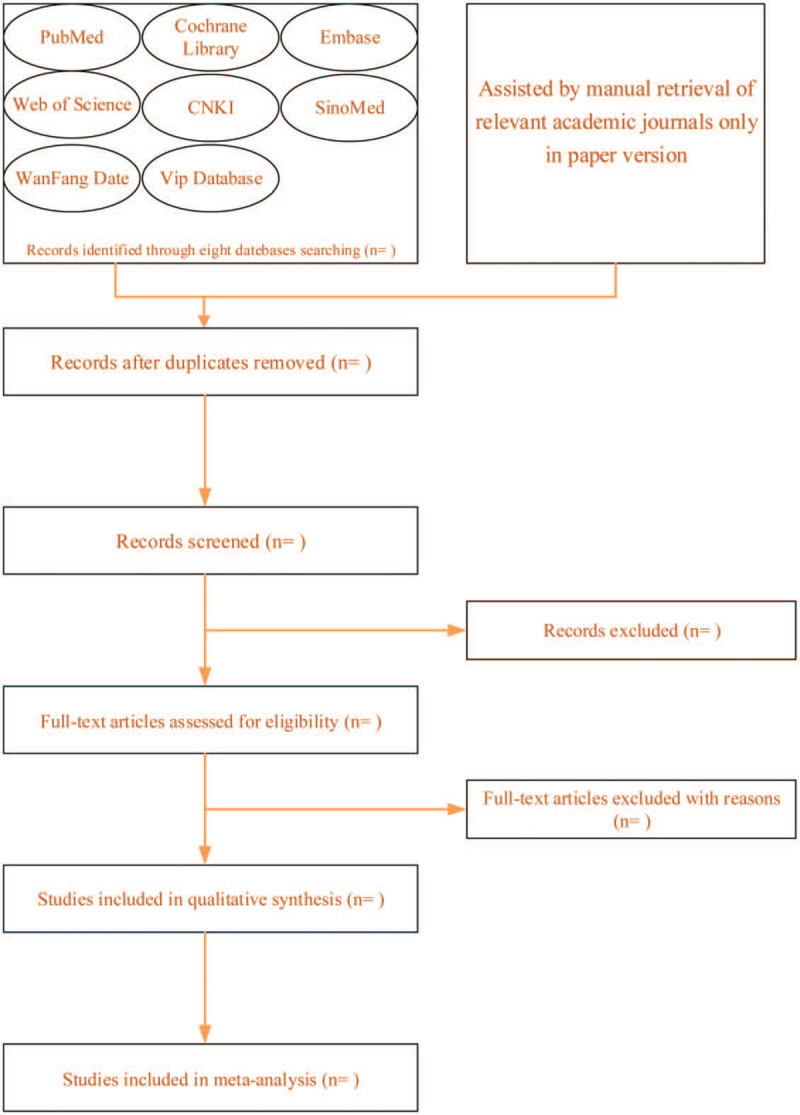
The process of literature screening by PRISMA flow diagram. PRISMA = Preferred Reporting Requirements for Systematic Review and Meta-Analysis.

According to the retrieval strategy, the 2 reviewers independently introduce the corresponding documents into the document management software, and finding out the duplicate literature to delete them, reading the title and abstract of the articles to delete the literature inconsistent with research (such as experience summary, review, and so on), and then reading the articles that basically meet the requirements. According to the inclusion and exclusion criteria, the qualified documents are extracted and crosschecked by the 2 reviewers based on the pre-designed data extraction form. In case of disagreement, the third party will judge to solve the problem. If the information and data are not comprehensive and unclear in the paper, contacting the original author to obtain relevant data information possibly. The process of literature screening will be shown by the Preferred Reporting Requirements for Systematic Review and Meta-Analysis flow diagram (Fig. 1).

### 3.6 Risk of bias (quality) assessment

Cochrane criteria are applied to assess the risk of RCT bias for inclusion, including^[18]^:

(1) whether the method of random allocation is detailed;
(2) whether the allocation is hidden and the method is perfect and correct;
(3) whether the blind method is applied;
(4) whether the result data are complete;
(5) whether the study results are selectively reported;
(6) whether other possible sources of bias are reported.

According to the Cochrane manual, judging the selected literature by referring to the above options. The result is that “yes” represents low bias risk, and “no” represents high bias risk, if the result is “no clear” represents a risk of uncertainty or unknown bias.

### 3.7 Statistical analysis

This meta-analysis applied RevMan 5.3 software to stat the related data, and continuous variables used mean difference, if the measurement indicators use different measurement methods or units are not uniform, standard mean difference will be used, and all studies are represented by 95% confidence interval. The heterogeneity of results is judged by *P* values and *I*^2^, if the test results are *P* < .1 or *I*^2^ > 50%, it can be considered that there is heterogeneity between groups. And the heterogeneity is large, using the random effect model to analyze, and conducting the sensitivity analysis to the results with greater heterogeneity; When *P* > .1 and *I*^2^ > 50%, it can be considered that the heterogeneity between groups is small, and the fixed effect model is used to analyze it. Forest plots is a tool to visualize the main contents of meta-analysis, when 95% confidence interval the horizontal lines intersect with the invalid lines, the incidence rate of observation group is equal to the incidence rate of the control group, and there is no statistical difference in the evidence body, whereas the evidence body is statistically different. Funnel plots use the therapeutic effect as the abscissa and the sample size as the ordinate to test publication bias by observing the symmetry of the scatter plot. At the same time, inverted funnel chart is used to detect publication bias for a research index with more than ten studies. If the inverted funnel plot is symmetrical, it indicates that there is no publication bias. Otherwise, it indicates that there may be bias.

### 3.8 Sensitivity analysis

Sensitivity analysis is also stability analysis, and conducting a new combined effect analysis by deleting a single study at a time, and comparing it with the meta-analysis before deletion to examine the impact of a single research result on the total effect amount, also checking whether the total effect is stable. If the difference between the 2 is large, it represents the sensitivity is high, the stability of the result is low, and the result is unreliable; otherwise, the sensitivity is low, the stability of the result is low, and the result is relatively reliable.^[19]^

### 3.9 Subgroup analysis

In the clinical study, due to different factors such as the basic information of the subjects, the severity of the disease, the dosage of drugs and the length of treatment course can affect the intervention effect, and when some of the reasons have a great influence on the results, the subgroup analysis under different factors can be carried out, so that the conclusion can be more clearly targeted.

### 3.10 Publication Bias

In this study, Rev Man 5.3 software was used to make the funnel plot. For small sample studies with large numbers and low accuracy, the effect estimates are mostly distributed at the bottom of the funnel plot and its distribution range is wider; for large sample studies with small numbers and high accuracy, the effect estimates are mostly distributed at the top of the funnel plot and its distribution range is wider.^[20]^ To observe whether the scattered points of the funnel plot are symmetrically distributed, it mainly determined by the size of the publication bias. When the number of studies in meta-analysis ≥10 original studies, it is generally recommended to do funnel plot to observe whether the funnel plot is symmetrical and asymmetric to evaluate the publication bias.

### 3.11 Ethics and dissemination

This study belongs to the category of the systematic review, which does not need ethical approval.

## 4 Discussion

RSV is the most common pathogen, which can cause viral pneumonia in children. There are 2 clinical types, the 1 is bronchiolitis, the other is interstitial pneumonia. This disease is prone to asthma, and serious cases will occur heart failure and threaten the lives of children.^[21]^ The disease rate of RSVP in infants and children is very high, and the maternal antibody has no obvious effect on the prevention of infection, so the disease rate of newborn babies is high. Combined with other factors, bronchiolitis and RSVP are the first among infants and children with viral pneumonia. In the process of clinical diagnosis and treatment, its clinical symptoms are not significantly different from mild adenovirus pneumonia, mild influenza virus pneumonia, parainfluenza virus pneumonia.^[22]^ The disease rate of RSV is very high, and it is prone to children under 2 years, its clinical symptoms are also difficult to distinguish with general bronchopneumonia. In addition to the characteristics of serious disease, it may also appear asthma, immune dysfunction and other symptoms, and seriously affect the quality of life in children. At present, it is often difficult to obtain the ideal therapeutic effect by using a single antiviral chemical drug in the course of clinical treatment for patients with RSV infection.^[23]^

The effects of Tanreqing on RSV in vitro showed that Tanreqing can protect cells from RSV invasion during prophylactic administration. It is speculated that the mechanism may be some molecules in Tanreqing, combining with HEp-2 cells or the corresponding parts of RSV surface, and changing its conformation or the number and structure of viral receptors on cell membrane to inhibit RSV penetration; during treatment and direct inactivation, the mechanism of Tanreqing inhibiting RSV proliferation and inactivation in vitro may be: Tanreqing could inhibit the replication of viral nucleic acid or prevent viral protein synthesis; some components of Tanreqing can combine with specific parts of RSV surface to make RSV inactive; Tanreqing injection stimulates the cell to secrete some molecular active substances, such as interleukin and interferon, to inhibit the proliferation of virus or kill directly, but its detailed mechanism needs further study.^[24,25]^ Ganciclovir is a widely used antiviral drug with high efficiency and broad-spectrum. The mechanism of ganciclovir inhibiting virus is as follows: on the 1 hand, it can directly penetrate into viral DNA and terminate the extension process of DNA replication; on the other hand, it competes to inhibit viral DNA polymerase and prevent DNA replication. After ganciclovir enters the host cell, it preferentially combines with the cell kinase of the host cell, and then is phosphorylated into ganciclovir triphosphate to block the growth of host cells and play a broad-spectrum antiviral effect under the induction of sensitive virus. Ganciclovir triphosphate has a high affinity with infected cells, and the drug concentration in infected cells is as high as 100 times. The half-life of ganciclovir is more than 24 hours, so it can maintain a long-term antiviral effect in infected host cells. In addition, ganciclovir has a certain selectivity to the virus. The concentration of ganciclovir in infected cells is 10 to 12 times higher than that in normal cells. Therefore, ganciclovir is safe and reliable and it will not affect normal human cells.

Because there are few reports on the treatment of RSVP in children with Tanreqing injection combined with ganciclovir at present, the safety and effectiveness of Tanreqing injection are uncertain, which leads doctors to dispute about its safety and effectiveness in the process of clinical application. Through this study, we will give the positive conclusion by using the scientific and rigorous research methods, and provide evidence-based medical evidence support for the effectiveness and safety of Tanreqing injection combined with ganciclovir on the treatment of RSVP in children, to provide assistance for clinical treatment.

## Author contributions

**Conceptualization:** Wei-Jun Zhu, Yu Shi.

**Data curation:** Xuan Zhou, Juan Cao.

**Formal analysis:** Wei-Jun Zhu.

**Funding acquisition:** Yu Shi.

**Investigation:** Wei-Jun Zhu, Xuan Zhou, Juan Cao.

**Methodology:** Wei-Jun Zhu.

**Software:** Xuan Zhou, Juan Cao.

**Supervision:** Wei-Jun Zhu.

**Writing – original draft:** Wei-Jun Zhu, Xuan Zhou, Juan Cao.

**Writing – review & editing:** Yu Shi.

## Abbreviations

Abbreviations: RSV = respiratory syncytial virus, RSVP = respiratory syncytial virus pneumonia.
